# Multimodal Human-Exoskeleton Interface for Lower Limb Movement Prediction Through a Dense Co-Attention Symmetric Mechanism

**DOI:** 10.3389/fnins.2022.796290

**Published:** 2022-04-25

**Authors:** Kecheng Shi, Fengjun Mu, Rui Huang, Ke Huang, Zhinan Peng, Chaobin Zou, Xiao Yang, Hong Cheng

**Affiliations:** ^1^School of Automation Engineering, University of Electronic Science and Technology of China, Chengdu, China; ^2^School of Mechanical and Electrical Engineering, University of Electronic Science and Technology of China, Chengdu, China; ^3^Glasgow College, University of Electronic Science and Technology of China, Chengdu, China; ^4^Department of Orthopedics, Sichuan Provincial People's Hospital, University of Electronic Science and Technology of China, Chengdu, China

**Keywords:** human-exoskeleton interface, lower limb movement prediction, multimodal, dense con-attention mechanism, hemiplegia rehabilitation training

## Abstract

A challenging task for the biological neural signal-based human-exoskeleton interface is to achieve accurate lower limb movement prediction of patients with hemiplegia in rehabilitation training scenarios. The human-exoskeleton interface based on single-modal biological signals such as electroencephalogram (EEG) is currently not mature in predicting movements, due to its unreliability. The multimodal human-exoskeleton interface is a very novel solution to this problem. This kind of interface normally combines the EEG signal with surface electromyography (sEMG) signal. However, their use for the lower limb movement prediction is still limited—the connection between sEMG and EEG signals and the deep feature fusion between them are ignored. In this article, a Dense con-attention mechanism-based Multimodal Enhance Fusion Network (DMEFNet) is proposed for predicting lower limb movement of patients with hemiplegia. The DMEFNet introduces the con-attention structure to extract the common attention between sEMG and EEG signal features. To verify the effectiveness of DMEFNet, an sEMG and EEG data acquisition experiment and an incomplete asynchronous data collection paradigm are designed. The experimental results show that DMEFNet has a good movement prediction performance in both within-subject and cross-subject situations, reaching an accuracy of 82.96 and 88.44%, respectively.

## 1. Introduction

Neurological diseases after spinal cord injury are one of the major causes of ipsilateral limb locomotion impairments, leading to functional disability and loss of independence in daily living activities for patients with hemiplegia (Langhorne et al., 2011). To reach better independence, early movement intervention in rehabilitation training is key to restoring motor function. The recent advances in a robotic exoskeleton have provided many technological solutions to movement intervention in rehabilitation training (Zimmermann et al., 2019; Lee et al., 2020; Keeling et al., 2021). Functional improvements have been reported in patients with hemiplegia (Chisari et al., 2015; Morone et al., 2018) after robot-aided rehabilitation training with a lower limb exoskeleton. In this context, it is of paramount importance to develop advanced human-exoskeleton interfaces to enhance the interaction between the wearer and the robotic exoskeleton and, thus, his active involvement, and maximizing the rehabilitation outcome (Lagoda et al., 2012; Gui et al., 2017; Lau et al., 2020).

Human-exoskeleton interface (HEI) is the core to enhance the interaction between the user and the robotic exoskeleton, and its main function is to predict the movement of exoskeleton wearers. Traditionally, HEI based on the physical signal, such as inertial measurement unit signal, is certainly one of the earliest used interfaces to predict the movements of the exoskeleton wearer (Beil et al., 2018; Zhu et al., 2020a). However, this kind of HEI makes low participation of patients in rehabilitation training. The rehabilitation outcome in the training process is not high. For this reason, biological neural signals [such as electroencephalogram and surface electromyography (sEMG)] have been introduced to the human-exoskeleton interface. This kind of HEI needs patients' active involvement. It uses patients' biological neural signals to predict their movement and usually achieves more effective rehabilitation outcomes.

The HEI based on biological neural signal is mainly divided into HEI based on sEMG and HEI based on electroencephalogram (EEG). Because of the strong correlation between sEMG signal and movement, the HEI based on sEMG signal has been earlier and most applied to robotic to help predict the movements of exoskeleton wearers (Lambelet et al., 2017; Yun et al., 2017; Wang et al., 2020). However, the usability of sEMG-based HEI strongly depends on the wearer's residual muscular functions. If the sEMG activity is highly affected by the limb's paresis, the prediction of the wearer's movement could be compromised by muscle weakness and early fatigue, making the use of these assistive HEI unfeasible on patients with hemiplegia (Millán et al., 2010). For this reason, the HEI based on EEG (also called brain-computer interface, BCI) is introduced into the robotic exoskeleton for predicting the movements of exoskeleton wearers. Some previous study has verified the feasibility of BCI in predicting movements of hemiplegic patients (Bhagat et al., 2016; Spüler et al., 2018; Dai et al., 2020). However, the EEG signal's signal-to-noise is low. It is susceptible to interfere with the environment and the patient's own limb movement and mood. These issues have strongly limited the diffusion of BCI in a broader population of patients and for applications outside a controlled laboratory environment.

The development of multimodal signal fusion technology urges people to use various biological neural signals to design HEI. HEI based on multimodal signals can realize the information complementarity between different modal data, and enhance the reliability of the HEI based on single-modal signals. These interfaces are usually fusion between sEMG and EEG signals, some studies have proved that the HEI based on multimodal signals can achieve a more accurate movement prediction (Li et al., 2017, 2020; Sarasola-Sanz et al., 2017; Nann et al., 2020; Tortora et al., 2020b). Previous studies have initially explored the feasibility of multimodal-based HEI in rehabilitation training, but there are some shortcomings: 1) There are few studies on the HEI for hemiplegic lower limb movement prediction. The work of predecessors mainly focused on the HEI based on hemiplegic upper limb movement prediction. 2) In previous study, sEMG signals and EEG signals were usually collected simultaneously (most studies focus on the upper limbs of hemiplegia, and upper limb movements have little interference with EEG signal collection). This kind of experimental paradigm interferes greatly with EEG signals during the collection of biologic signals of lower limb movement, which will affect the reliability of the HEI. 3) In the research of HEI based on multimodal signal fusion, most HEI just simply merge the movement prediction results or data channels of different modal signals. Few studies have considered the fusion of sEMG and EEG signal features, let alone the deep connection between sEMG and EEG signal features.

In response to the above problems, this article proposes a multimodal enhanced fusion human-exoskeleton interface for lower-limb hemiplegia movement prediction. This interface uses a dense co-attention (DCA) mechanism to achieve the mapping and deep fusion between sEMG and EEG signal features. The main contributions are as follows:

- An experimental paradigm for the incomplete asynchronous acquisition of EEG and sEMG signals is designed, which solves the problem of large interference to EEG signals from the synchronous acquisition of EEG and sEMG signals of the hemiplegic lower limbs.
- Aiming at the high accuracy movement prediction of the lower limbs of hemiplegia, a DCA mechanism based Multimodal Enhance Fusion Networks (DMEFNet) is proposed, which realizes the mapping and deep fusion between the sEMG signal and the EEG signal feature.
- In the two cases of within-subject and cross-subject, the lower limb movement prediction performance of DMEFNet is compared with six traditional machine learning-based movement prediction models and two deep learning-based movement prediction models.

## 2. Related Study

Human-exoskeleton interface is a kind of Human-robot interface (HRI). According to different biological signals, HRI can be divided into HRI based on sEMG signal (MHRI) and HRI based on EEG (BCI).

### 2.1. Related Work of MHRI-Based Movement Prediction

As the biological signal most relevant to exercise, sEMG has been applied to HRI for a long time, and the research on MHRI is particularly rich. An entire MHRI includes three main processing stages data collection and processing, feature extraction, and classification. Traditional MHRI mainly extracts some manual normative time-domain, frequency-domain, and time-frequency domain features, and then uses machine learning methods to construct the mapping between features and different movements (Yun et al., 2017; Cai et al., 2019; Wang et al., 2020). Literature (Yun et al., 2017) proposed an ANN-based upper limb movement prediction model. This model uses the sEMG signals of three muscles from the hemiplegic patients' forearms to predict the movements of the upper limbs of the patients, which can achieve more accurate movement prediction. Cai et al. (2019) proposed an SVM-based upper limb movement prediction method, which uses the sEMG signal of the unaffected upper limb muscles of the hemiplegic patient to predict the movement of the patient's shoulder and elbow joints, with an accuracy of 93.56%.

Deep learning can automatically extract the best feature set from sEMG signals. Many researchers have explored the application of deep learning in MHRI-based movement prediction methods (Allard et al., 2016; Côté-Allard et al., 2019). Allard et al. (2016) proposed a multi-layer CNN gesture prediction model based on sEMG for robot guidance tasks. The model automatically extracts the frequency domain features of different gesture movements through the CNN architecture, and the average accuracy of gesture prediction for 18 subjects is 93.14%. Considering that the movement prediction model based on deep learning usually requires a lot of data, this will generate a lot of labor cost and time cost. Côté-Allard et al. (2019) proposed a gesture prediction model based on transfer learning, which uses the sEMG signals of 17 subjects to achieve an average accuracy of 98.53%.

### 2.2. Related Work of BCI-Based Movement Prediction

A brain-computer interface (BCI) enables direct communication with a robot *via* EEG signals (Wolpaw et al., 2002). Traditionally, BCI is mainly used in the field of medical rehabilitation to realize the perception of user intent. BCI also includes the same three data processing stages (Lotte et al., 2018), and unlike MHRI, BCI based on traditional machine learning methods mainly extracts some manual normative spatial domain features (Lee et al., 2019), and then uses machine learning methods to construct the mapping between spatial domain features and different movements. Literature (Wang et al., 2017) used the common space pattern (CSP) model to extract the spatial features of the subject's motor imagery (MI) EEG signals and then designed a support vector machine-based BCI to realize accurate prediction of lower limb movements.

Recent research has explored the application of deep learning in BCI (Gao et al., 2020; Tortora et al., 2020a). Tortora et al. (2020a) proposed a gait pattern prediction method based on an LSTM architecture. This method uses the LSTM construct to automatically extract and classify the timing features of the EEG signal, which can achieve an accuracy of 92.8%. Gao et al. introduced a deep learning method to cope with the 'BCI Illiterate' phenomenon, in which case some subjects present unsatisfactory performance with low classification accuracies. They constructed a convolutional neural network with long short-term memory (CNN-LSTM) framework, where the network allows extracting the spectral, spatial, and temporal features of EEG signals. The results show that the DL method can reach 91.86% for EEG illiterate subjects (Gao et al., 2020).

### 2.3. Related Work of HRI Based on Multimodal Signals

Human-robot interface based on multimodal signals has more or fewer limitations, so recent attempts to overcome the limitations of single-modal signal-based HRI have brought forward approaches combining multiple HRIs, including at least one BCI integrated with other BCIs or other biologic signals (e.g., sEMG and electrooculogram). Zhu et al. (2020b) used the combination of EEG and electrooculogram (EOG) signals to realize the grasping and moving tasks of the robotic arm, with an average accuracy of 92.09%. Literature (Tortora et al., 2020b) proposed a hybrid HRI to predict walking phases of both legs from the Bayesian fusion of EEG and sEMG signals, the HRI significantly outperforms its single-signal counterparts, by providing high and stable performance even when the reliability of the sEMG signal activity is compromised temporarily. However, the previous study simply spliced signals or signal features of different modes together. These works did not consider the deep connection and fusion between different modal signals, which resulted in poor robustness of HRI movement prediction performance, especially in the case of cross-subjects.

With the research of attention mechanisms, some researchers try to use attention mechanisms to fuse essential features of different modal signals to improve the classification performance of HRI (Khushaba et al., 2020; Tao et al., 2020; Zhang et al., 2021; Zhao and Chen, 2021). Zhang *et al*. used a multi-dimensional feature fusion network framework to detect muscle fatigue, which used a time-domain attention network and a frequency-domain attention network to extract important time-domain features and frequency-domain features in sEMG signals, respectively, and then Feature fusion is performed, and the results show that the proposed framework can effectively improve the detection performance of muscle fatigue (Zhang et al., 2021). After extracting the time-series features of EEG data in different frequency bands, Zhao et al. used the self-attention mechanism to extract the essential features of EEG signals in different frequency bands to extract the accuracy of HRI in emotion recognition (Zhao and Chen, 2021). These studies demonstrate that the attention mechanism can extract important components between different modal signal features and improve the classification performance of HRI. However, it does not consider the correlation between different modal features and the parts that concern each other.

The co-attention mechanism is a popular network structure in the field of computer vision in the past 2 years. It is widely used in cross-modal retrieval research to find common areas of interest between data features of different modalities, thereby enhancing the fusion of data features (Nguyen and Okatani, 2018; Yu et al., 2019). In the study of this article, we introduced a co-attention mechanism to design the human-exoskeleton interface for the lower limb movement prediction of patients with hemiplegia. This interface uses the co-attention mechanism to achieve deep fusion between sEMG and EEG signal features, which can improve the lower limb movement prediction performance of the human-exoskeleton interface.

## 3. Method

The goal of this article is to design a human-exoskeleton interface for hemiplegic lower limb rehabilitation training. Its core method is to use sEMG and EEG signal data to predict the movements of the lower limbs. This section presents the methodology details of the proposed movement prediction model.

### 3.1. Overview of the DMEFNet Model

**Figure 1:**
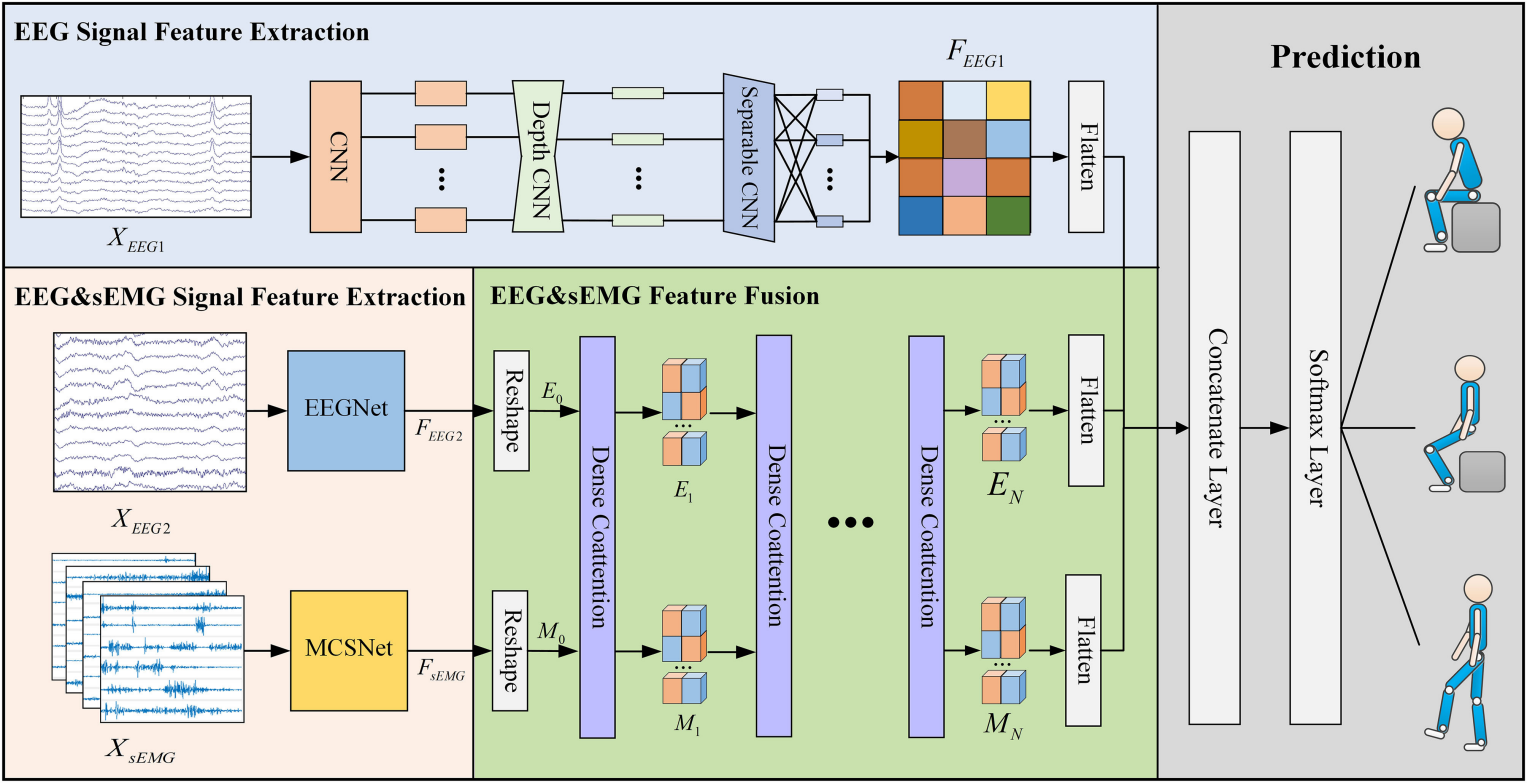
The overall architecture of the DMEFNet model. This model contains two feature extraction modules, a feature fusion module, and a lower limb movement prediction module. The feature extraction module uses EEGNet and MCSNet to extract the features of EEG signals and sEMG signals, respectively. The feature fusion module enhances the co-attention between sEMG and EEG signal features through the DCA mechanism.

Figure 1 visualizes the proposed DMEFNet movement prediction model. Since this paper designs an incomplete asynchronous sEMG and EEG signal acquisition experimental paradigm (refer to section 4.1 and **Figure 3** for details), the input of the model is divided into two parts: the first part is the asynchronous (individually) collected motor imagery EEG (MI-EEG) data ($X_{EEG1}\in {ℜ}^{C_{1}\times T_{1}}$), and the second part is the synchronously collected sEMG data ($X_{sEMG}\in {ℜ}^{C_{2}\times T_{2}}$) and EEG data ($X_{EEG2}\in {ℜ}^{C_{1}\times T_{2}}$). According to the data input and feature extraction methods, the entire model architecture consists of four parts. The first part is the feature extraction of *X*_*EEG*1_, using the EEGNet (Lawhern et al., 2018) to extract the features of the first part of the MI-EEG data. The second part is the feature extraction of *X*_*EEG*2_ and *X*_*sEMG*_, where the features of sEMG data are extracted using the previous study's MCSNet (Kecheng et al., 2021), and the second part of the MI-EEG data is extracted using the EEGNet model. The third part is DCA symmetric network (Nguyen and Okatani, 2018). It is used to obtain the fusion feature representation of the data *X*_*EEG*2_ and *X*_*sEMG*_. The fourth part is movement classification/prediction, which classifies the features extracted from the first and third parts.

### 3.2. EEG Signal Feature Extraction

In the DMEFNet model, for the asynchronously collected MI-EEG data *X*_*EEG*1_ and the synchronously collected MI-EEG data *X*_*EEG*2_, the EEGNet model is used for feature extraction. EEGNet (Lawhern et al., 2018) is a compact BCI model proposed by Lawhern. It has good generalization ability for EEG signals collected under different paradigms, and can also achieve good classification performance for small-scale data. The entire EEGNet mainly contains two modules, the first module contains a CNN layer and a depthwise CNN layer. The CNN layer is mainly used to learn the frequency domain features of EEG signals in different frequency bands, and the depthwise CNN layer is to learn the spatial features of different frequency domain features. The second module of EEGNet is a separable CNN layer, which is used to combine and optimize the features between different channels. After passing the EEGNet model, the features of EEG data *X*_*EEG*1_ and *X*_*EEG*2_ can be expressed as:


(1) $$\begin{array}{r} \begin{array}{cc} F_{EEG1} & =EEGNet\left(X_{EEG1}\right),\\ F_{EEG2} & =EEGNet\left(X_{EEG2}\right), \end{array} \end{array}$$

### 3.3. sEMG Signal Feature Extract

For the synchronously collected sEMG data *X*_*sEMG*_, this article uses the previous work MCSNet to perform feature extraction on the *X*_*sEMG*_ data. MCSNet contains a total of 3 modules. The first module is an LSTM layer, which is used to extract the time domain features of the sEMG signal channel by channel. The second module is a two-layer CNN network. It extracts the frequency domain features under the time domain features of different channels (that is, the time-frequency features of different channels of the sEMG signal). The third module of MCSNet is a Depthwise CNN layer, which is used to combine and optimize the time-frequency features between different channels. Namely, it is used to extract the synergy features between the sEMG signal channels. Same as above, the feature extracted from *X*_*sEMG*_ data can be expressed by Equation 2.


(2) $$\begin{array}{r} F_{sEMG}=MCSNet\left(X_{sEMG}\right), \end{array}$$

### 3.4. DCA Symmetric Network

**Figure 2:**
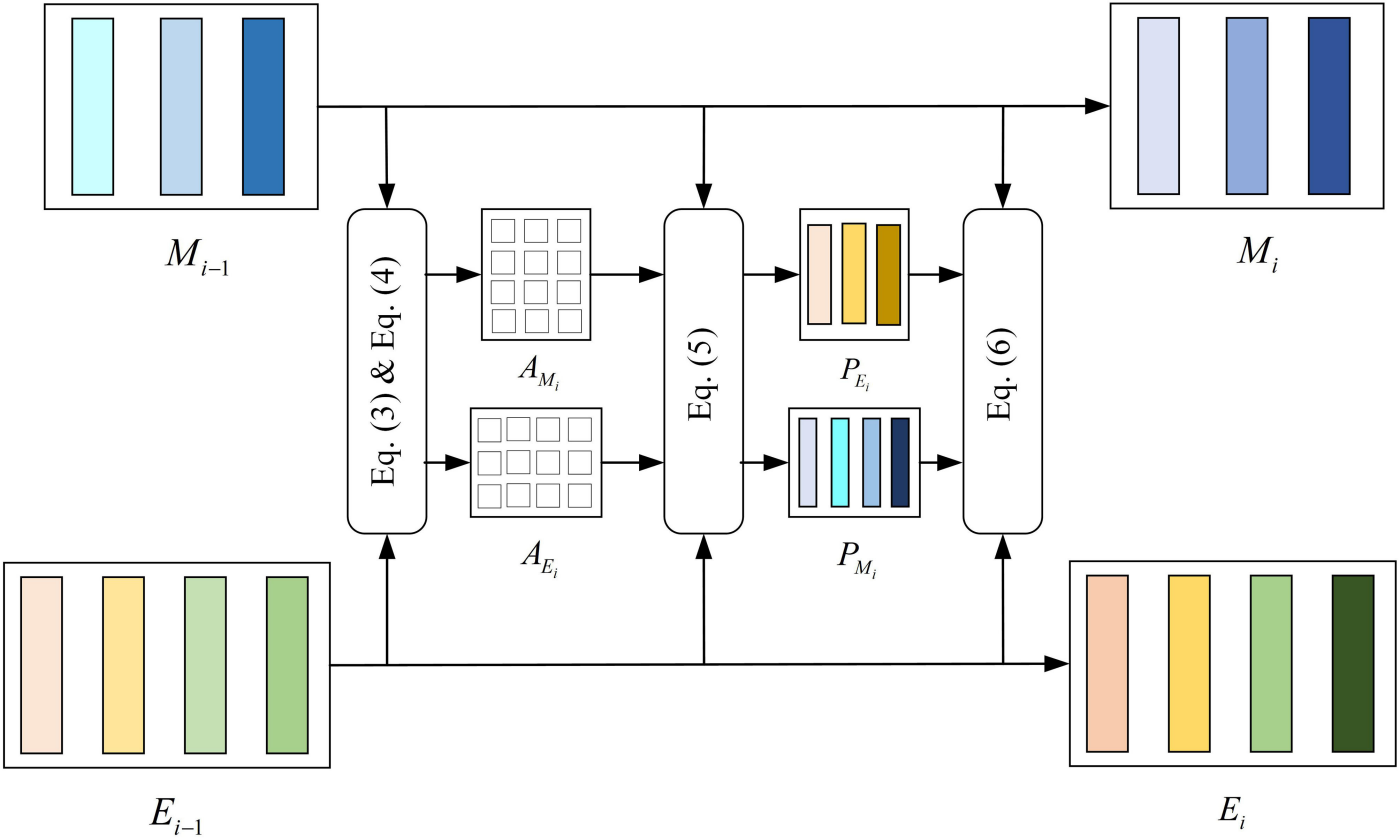
Structure of the dense co-attention (DCA) layer.

Dense Co-Attention Symmetric Network (DCAN) was originally proposed to solve the visual question answering (VQA) problems. It presents an architecture that enables dense and bidirectional interactions between two modalities and contributes to boosting the prediction accuracy of answers (Nguyen and Okatani, 2018). The network contains three main parts: feature extraction, stacked DCA layers, and prediction. In the DMEFNet model, we only use the stacked DCA layers of DCAN to achieve the mapping and deep fusion between sEMG and EEG signal features (as shown in Figure 2).

Since Stacked DCA Layers requires the input of the two modal data to have the same length in the feature representation dimension, we performed the reshape operation on the simultaneously collected sEMG and EEG signal features after feature extraction. The sEMG signal feature after the reshape operation, *M*_0_, is sized to (*d*×*n*_*m*_), and the EEG signal feature after the reshape operation, *E*_0_, is sized to (*d*×*n*_*e*_). *M*_0_ [*M*_0_ = *reshape*(*F*_*sEMG*_)] and *E*_0_ [*E*_0_ = *reshape*(*F*_*EEG*2_)] are fed into the DCA layer directly.

The DCA layer does not change the dimensions of the input and output. For the DCA (*i*)-st layer, the output from the previous layer *M*_*i*−1_ and *E*_*i*−1_ are first interacted by multiplication with a learnable weight matrix *W*_*i*_ and get the affinity matrix of sEMG and EEG signal (refer to the Equation 3).


(3) $$\begin{array}{r} A_{i}={E_{i-1}}^{T}W_{i}M_{i-1}, \end{array}$$

where $A_{i}\in {ℜ}^{n_{e}\times n_{m}}$, $W_{i}\in {ℜ}^{d\times d}$ is a learnable weight matrix.

Then we normalize *A*_*i*_ row-wise to derive attention maps on sEMG features conditioned by each EEG feature and also normalize *A*_*i*_ column-wise to derive attention maps on EEG features conditioned by each sEMG feature, as shown in Equation 4.


(4) $$\begin{array}{r} \begin{array}{cc} A_{M_{i}} & =softmax\left(A_{i}\right),\\ A_{E_{i}} & =softmax\left(A_{i}^{T}\right), \end{array} \end{array}$$

Next, *M*_*i*−1_ and *E*_*i*−1_ interact by multiplying *A*_*M*_*i*__ and *A*_*E*_*i*__, respectively, obtaining *P*_*M*_*i*__ and *P*_*E*_*i*__. Finally, the result of the DCA (*i*)-st layer, *M*_*i*_ and *E*_*i*_ are computed after the interaction by concatenating *M*_*i*−1_ to *P*_*E*_*i*__, *E*_*i*−1_ to *P*_*M*_*i*__ (refer to the Equations 5 and 6). The features of sEMG and EEG have been further integrated.


(5) $$\begin{array}{r} \begin{array}{cc} P_{M_{i}} & =M_{i-1}A_{M_{i}}^{T},\\ P_{E_{i}} & =E_{i-1}A_{E_{i}}^{T}, \end{array} \end{array}$$

(6) $$\begin{array}{r} \begin{array}{cc} M_{i} & =ReLU\left(W_{M_{i}}\left[\begin{array}{c} M_{i-1}\\ P_{E_{i}} \end{array}\right]+b_{M_{i}}\right)+M_{i-1},\\ E_{i} & =ReLU\left(W_{E_{i}}\left[\begin{array}{c} E_{i-1}\\ P_{M_{i}} \end{array}\right]+b_{E_{i}}\right)+E_{i-1}, \end{array} \end{array}$$

where *W*_*M*_*i*__, *W*_*E*_*i*__∈ℜ(*d*×2*d*) and *b*_*M*_*i*__, *b*_*M*_*i*__∈ℜ(*d*×1) are learnable weight matrix and bias.

After the calculation through the stacked DCA layers, we flatten the outputs of the DCA layer and the asynchronously collected EEG signal feature *F*_*EEG*1_, and they are followed by a softmax operation. Finally, we can get the predicted lower limb movement. It can be expressed by Equation 7.


(7) $$\begin{array}{r} Label_{predicted}=Softmax\left(Flatten\left(F_{EEG1},M_{N},E_{N}\right)\right). \end{array}$$

where *Label*_*predicted*_ is the label of lower-limb movement predicted, N is the number of DCA layers.

### 3.5. Comparison With Other Movement Prediction Approaches

In terms of movement prediction models based on traditional machine learning, we compare the performance of DMEFNet with six traditional movement prediction models based on handcrafted features. In the selection of sEMG signal features, referring to the research conclusions of time domain and frequency domain features in the literature (Phinyomark et al., 2012), we finally selected the feature of Mean Absolute Value (MAV), WaveLength (WL), Zero Crossings (ZC), 6-order Auto-Regressive coefficient (6-AR), and average Power Spectral Density (PSD). As for EEG signal features, we chose the most commonly used CSP features. Linear Discriminant Analysis (LDA), Decision Tree (DT), Naive Bayes (BES), Linear Kernel-based Support Vector Machine (LSVM), Radial Basis Function-based Support Vector Machine (RBFSVM), K Nearest Neighbor (KNN), and Artificial Neural Network (ANN) are chosen as the classification/prediction model. All the traditional movement prediction models are implemented by MATLAB's Classification Learner Toolbox and Neural Net Pattern Recognition Toolbox.

In terms of deep learning, we compared the performance of DMEFNet with EEGNet and our previous study MCSNet. The specific structure has been described in Section 3. We implemented these models in PyTorch. For specific details of the model, see https://github.com/mufengjun260/DMEFNet.

## 4. Experiments and Results

In this part, an sEMG and EEG signal incomplete asynchronous acquisition experiment is designed to verify the effectiveness of the method proposed in this manuscript. Section 4.1 describes the acquisition experiment process and data preprocessing methods. Section 4.2 gives the implementation details of model training. In Section 4.3, we show the DMEFNet movement prediction model results and compare DMEFNet with other movement prediction models in the case of within-subject and cross-subject.

### 4.1. sEMG and EEG Data Acquisition Experiment

**Figure 3:**
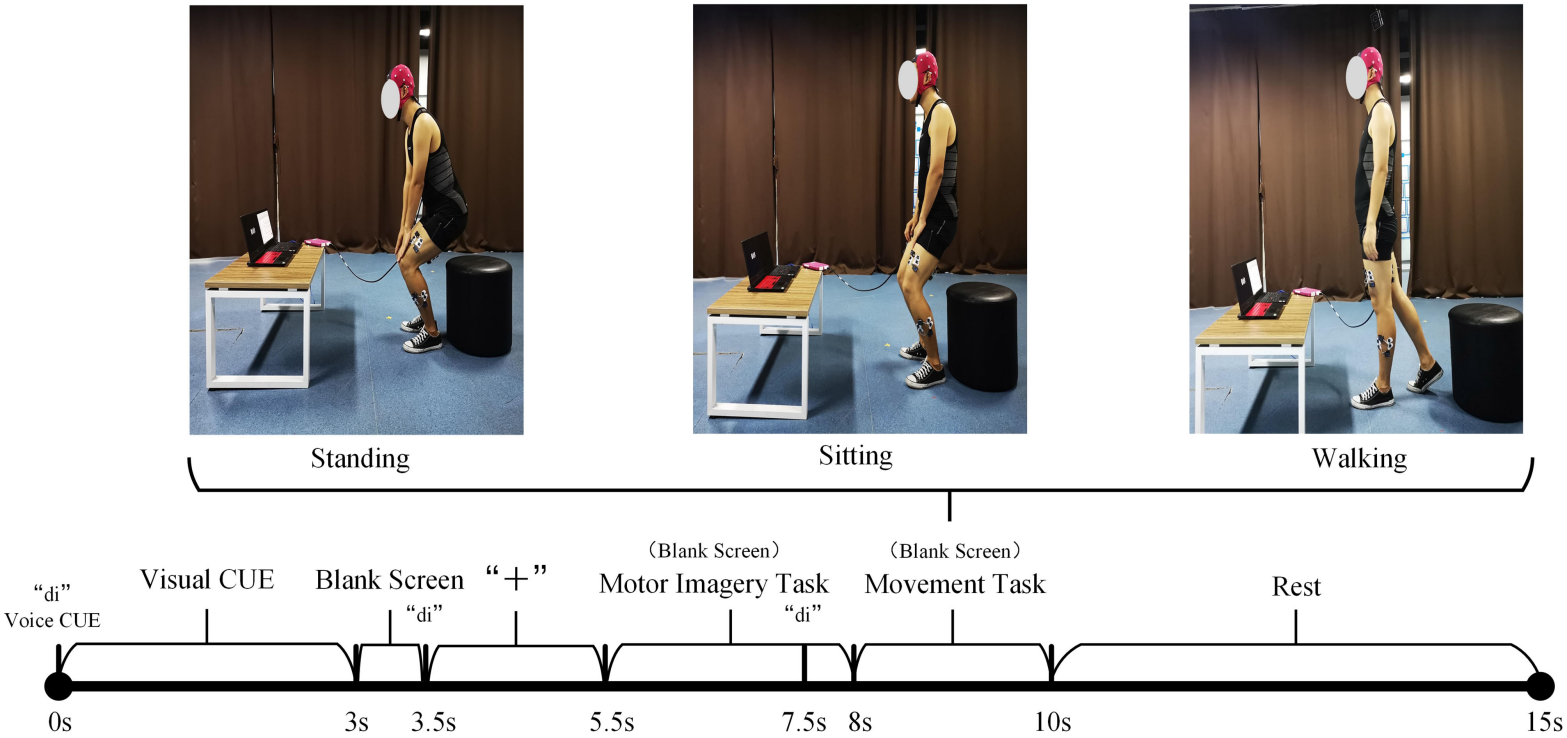
The incomplete asynchronous acquisition paradigm of the sEMG and EEG data acquisition experiment. The upper part is the three lower limb movements. The lower part is the incomplete asynchronous acquisition paradigm of sEMG and EEG data. As can be seen from the figure, EEG signals and sEMG signals are not collected completely asynchronously. After 7.5s, the EEG signal and sEMG signal are collected simultaneously. Considering that the sEMG signal will be activated within 0.5–1 s before the action is performed, and the subject needs a period of time to respond to the sound prompt, this paper believes that the sEMG signal and EEG signal collected simultaneously in 7.5–8.5 s have an important role in the prediction of the lower limb movement of hemiplegia. According to the definition in Section 3.1, *X*_*EEG*1_ is the EEG signal data collected in the period of 5.5–7.5s. *X*_*EEG*2_, *X*_*sEMG*_ are the EEG and sEMG signal data collected simultaneously during the 7.5–8.5 period, respectively.

A total of 10 healthy subjects are invited to participate in the experiment. Considering that the synchronous acquisition of sEMG and EEG signals during the movement of the lower limbs will cause a great interference to the EEG signals, an experimental paradigm is proposed for the incompletely asynchronous acquisition of sEMG and EEG signals (as shown in Figure 3). Under this paradigm, each subject completed the motor imagery task under the three movements of standing, sitting, and walking and performed these movements. The sEMG signals of subjects' lower limb muscles and EEG signals are collected during this period.

#### 4.1.1. Participants

The 10 subjects (7 men, 3 women) have an average age of 24 years, a height between 160 and 180 *cm*, and a weight between 50 and 82 *kg*. All subjects can independently complete the lower limb movements involved in the experiment, and they are in good physical and mental condition with no injuries to the lower limbs. Before the experiment, each subject had been explained the contents of the experiment and signed an informed consent form. This experiment was approved by the Research Ethics Committee of the University of Electronic Science and Technology of China.

#### 4.1.2. Procedures

**Figure 4:**
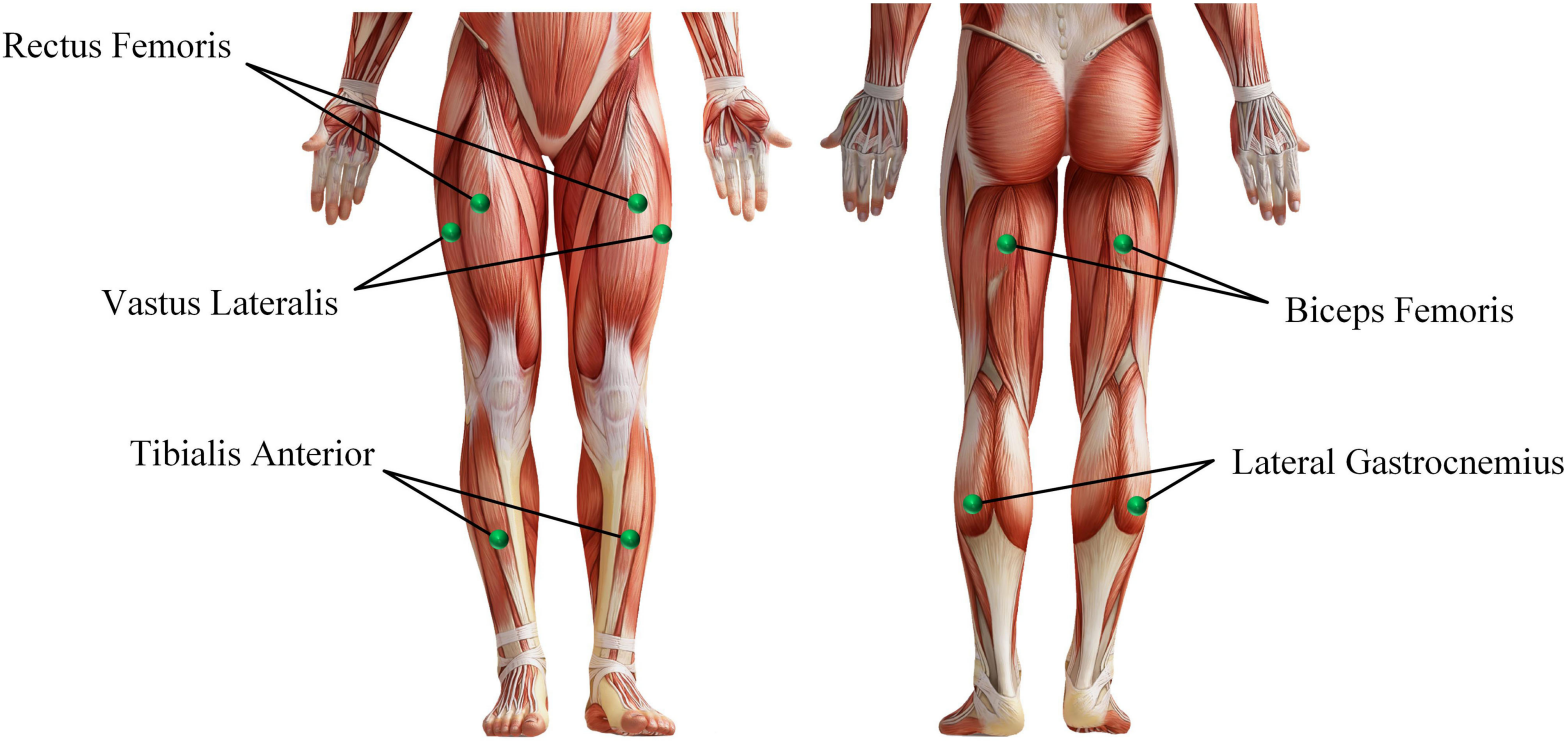
The muscle used in the sEMG and EEG data acquisition experiment.

Before the experiment, record the relevant physical parameters of the subject, and inform the experimental procedure to the subject. Then let the subject wear an EEG cap, apply electrode gel, and paste sEMG acquisition electrodes on the 10 muscles of the left and right legs (the rectus femoris, vastus lateralis, tibialis anterior, biceps femoris, and lateral gastrocnemius of every leg, as shown in Figure 4). During the experiment, the subjects naturally relax, standing in front of the computer screen, and then follow the incomplete asynchronous acquisition experimental paradigm to perform the corresponding actions (first perform motor imagery task, and then perform the corresponding lower limb movements), details as follows:

- 0–3s: The computer emits a “di” prompt. A video about lower limb movement is played on the screen, reminding subjects of the movement to perform MI tasks.
- 3–5.5s: The screen is in a black screen state. At 3.5s, the computer emits a “di” prompt for the second time and a “+” pattern appears on the screen, prompting the subject to prepare for the MI task. The “+” pattern lasts for a total of 2s, and then the computer screen returns to a black screen state.
- 5.5–8s: The screen is in a black screen state, and the subject is performing a MI task. The MI task lasted 2.5s. At 7.5s, the computer emits a "di" prompt for the third time to remind the subject to prepare to perform the corresponding lower limb movement.
- 8–10s: The screen is in a black screen state, and the subject performs the corresponding lower limb movement. The whole movement is completed within 2s.
- 10–15s: The “○” pattern appears on the screen, reminding the subject that the single-movement data collection experiment is over. The subjects rest and return to the position and posture at the beginning of this experiment, waiting for the next experiment.

There are three groups in the whole experiment, and each group contains 30 lower limb movements, of which 10 are standing, sitting, and walking. The walking and sitting movements appear randomly in the first 20 movements, and the last 10 movements are standing movements (Because performing a sitting or walking movement requires the subject to be in a standing position, and performing a standing movement requires the subject to be in a sitting position. This also helps subjects perform MI tasks). During the whole experiment, myoMUSCLE (an sEMG acquisition device, Scottsdale, and American) and waveguard (an EEG acquisition device, Hengelo, and The Netherlands) are used to record the subject's lower-limb sEMG signal and MI EEG signal data.

#### 4.1.3. Data Processing

myoMUSCLE (1,500 *Hz*, 10 channels) collects the sEMG signal data of each lower limb movement of the subject, and waveguard (1,000 *Hz*, 32 channels) collects the EEG signal data of each MI of the subject. After obtaining the sEMG data, a 50 *Hz* notch filter is used to remove the power frequency interference of the current, and a 10–450 *Hz* bandpass filter is used to retain the effective information of the sEMG signal. As for the EEG data, a 0.3–30 *Hz* band-pass filter is used to retain the effective information of the MI EEG signal. Since the application of this article is aimed at the prediction of lower extremity hemiplegia rehabilitation training (the subject first performs MI, and then performs lower extremity movement prediction), this article only uses the EEG signal data during the period of 5.5−8.5*s* and the sEMG signal during the period of 7.5−8.5*s*. Considering the availability of biological nerve signal data on the affected side of the hemiplegia patient, we only take the sEMG signal channel and EEG signal channel of the subject's one-side body (i.e., 5 sEMG signal channels + 16 EEG signal channels) to train the proposed model. In addition, for the sEMG signal, this article uses 200 *ms* (including 300-time series data) as a time window to segment the sEMG signal, and the movement step of the time window is 100-time series data (Yu et al., 2015; Shen et al., 2019).

### 4.2. Implementation Details

DCA mechanism based Multimodal Enhance Fusion Networks is implemented using the PyTorch library (Paszke et al., 2017). In DMEFNet, the network's hyper-parameters (*C*_1_, *C*_2_, *T*_1_, *T*_2_) are set to (14, 2,000, 5, 1,500), and all LSTM's output and hidden unit are of dimension 300. Exponential linear units (ELU) (Clevert et al., 2015) are used to introduce the nonlinearity of each convolutional layer. To train our model, we use the Adam optimizer to optimize the model's parameters, with the default setting described in Kingma and Ba (2014) to minimize the categorical cross-entropy loss function. We run 1,000 training iterations (epochs) and perform validation stopping, saving the model weights, which produces the lowest validation set loss. All models are trained on NVIDIA RTX2080Ti, with CUDA10.1 and cuDNN V7.6. Our parameter settings and code implementation can be found at https://github.com/mufengjun260/DMEFNet.

### 4.3. Experiment's Result and Discussion

We compared the performance of the proposed DMEFNet model with six traditional machine learning-based movement prediction models and two deep learning-based movement prediction models in movement classification/prediction in both the within-subject and cross-subject situations.

#### 4.3.1. Within-Subject Classification

For within-subject, we divide the data of the same subject according to a ratio of 7:3 and then use 70% of the data to train the model for that subject. Four-fold cross-validation is used to avoid the phenomenon of model overfitting. Simultaneously, repeated-measures analysis of variance (ANOVA) is used to test the results statistically (using the number of subjects and the classification model as factors, and the model classification/prediction result (accuracy) as the response variable).

**Figure 5:**
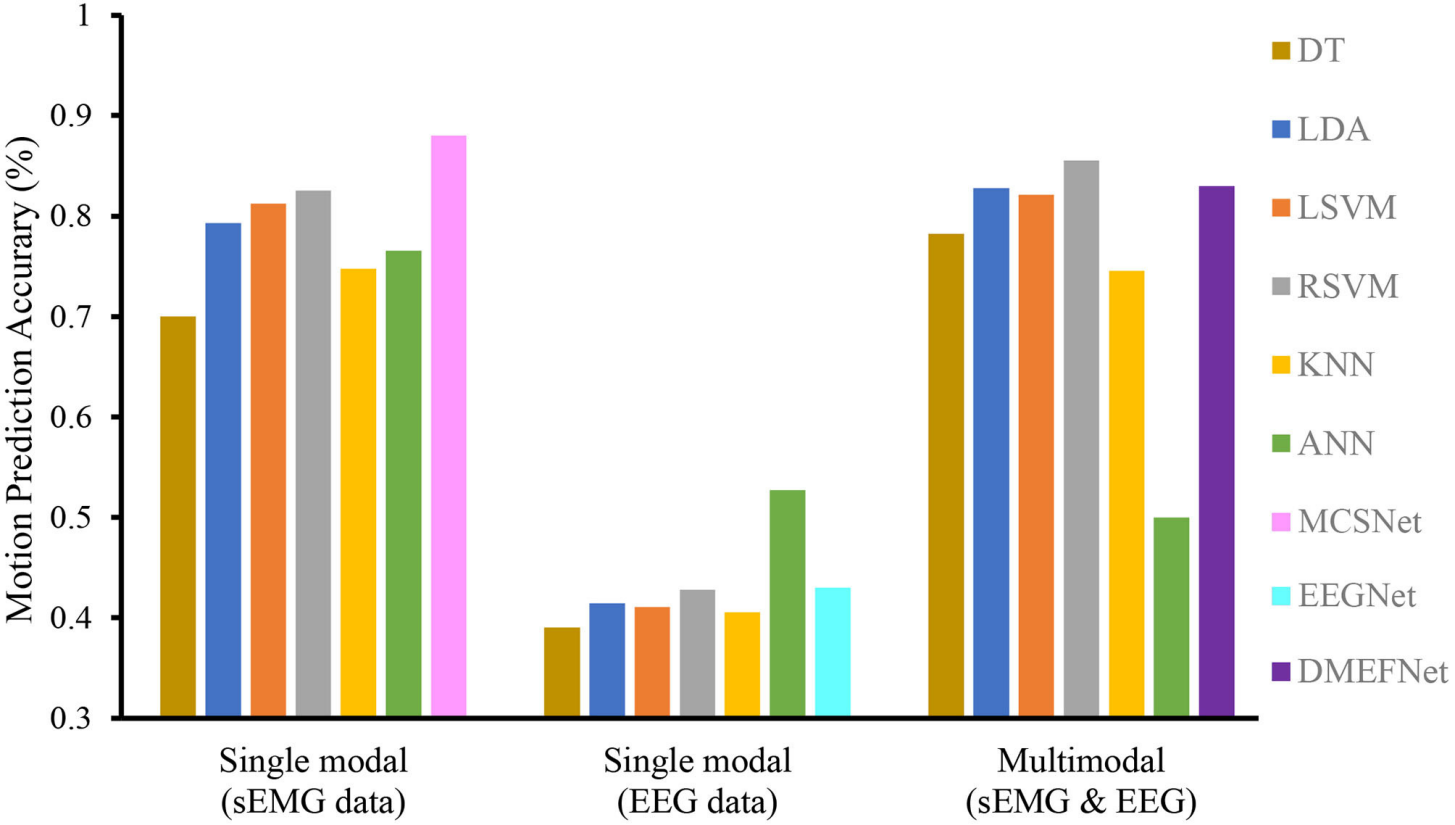
Within-subject movement prediction performance, the input of six traditional machine learning-based movement prediction models are sEMG and EEG multimodal data. Four-fold cross-validation is used to avoid the phenomenon of model overfitting, averaged over all folds and all subjects. Error bars denote two standard errors of the mean.

We compare the performance of traditional machine learning-based movement prediction models (LDA, DT, BES, LSVM, RBFSVM, KNN, and ANN) with DMEFNet. Within-subject results across all models are shown in Figure 5. From the figure, we can clearly observe that in the case of single-modal data, the accuracy of the movement prediction model based on the sEMG signal is much greater than that of the movement prediction model based on EEG signal, which shows that the sEMG signal contains more information related to lower limb movement. Second, the accuracy of lower limb movement prediction based on multimodal signals is better than that based on single-modal signals, which shows that the combination of sEMG and EEG signals can complement information and enhance the performance of the movement prediction model. Furthermore, it can be clearly found that, in the two cases of single modal data and multimodal data, the performance of DMEFNet proposed in this article for lower limb movement prediction is not the best compared to other movement prediction models. However, its performance in movement prediction is close enough to the best movement prediction models, such as MCSNet using sEMG single-modal data and RBFSVM using multimodal data. It can be seen intuitively from Table 1 that their performance gap is less than 5%. DMEFNet contains an attention mechanism and deep learning network framework, while a single subject itself contains not much training data. In this case, the movement prediction performance of the model is close enough to the performance of the machine learning-based movement prediction model, indicating that DMEFNet can also extract effective fusion features in the case of small sample data, which proves that DMEFNet is effective.

**Table 1:** Within-subject movement prediction performance (Test set ACC).

**Lower limb movement prediction accuracy of test set**												
	**Method**	**1**	**2**	**3**	**4**	**5**	**6**	**7**	**8**	**9**	**10**	**AVE ACC**
Single modal (sEMG data)	LDA	0.79	0.79	0.89	0.78	0.82	0.67	0.79	0.79	0.87	0.74	**0.79**
	DT	0.60	0.61	0.77	0.77	0.77	0.57	0.75	0.67	0.74	0.76	**0.70**
	LSVM	0.84	0.81	0.92	0.77	0.81	0.73	0.80	0.80	0.88	0.76	**0.81**
	RSVM	0.84	0.78	0.91	0.86	0.83	0.74	0.82	0.82	0.90	0.75	**0.83**
	KNN	0.78	0.75	0.83	0.76	0.75	0.65	0.74	0.75	0.77	0.69	**0.75**
	MCSNet	0.85	0.93	0.96	0.93	0.93	0.59	0.93	0.89	0.96	0.81	**0.88**
Single modal (EEG data)	LDA	0.34	0.39	0.46	0.41	0.38	0.31	0.40	0.44	0.52	0.49	**0.41**
	DT	0.39	0.37	0.37	0.49	0.36	0.30	0.34	0.35	0.45	0.48	**0.39**
	LSVM	0.35	0.38	0.45	0.44	0.36	0.32	0.36	0.44	0.51	0.49	**0.41**
	RSVM	0.36	0.42	0.44	0.44	0.39	0.32	0.39	0.48	0.52	0.51	**0.43**
	KNN	0.37	0.35	0.43	0.51	0.36	0.30	0.36	0.46	0.50	0.42	**0.41**
	EEGNet	0.41	0.33	0.44	0.33	0.52	0.44	0.48	0.52	0.48	0.37	**0.43**
Multimodal (sEMG & EEG)	LDA	0.84	0.81	0.91	0.81	0.82	0.75	0.86	0.78	0.88	0.81	**0.83**
	DT	0.80	0.77	0.86	0.76	0.73	0.68	0.86	0.77	0.85	0.75	**0.78**
	LSVM	0.80	0.79	0.93	0.83	0.81	0.72	0.88	0.77	0.84	0.86	**0.82**
	RSVM	0.85	0.84	0.92	0.88	0.83	0.74	0.89	0.84	0.88	0.87	**0.86**
	KNN	0.82	0.73	0.82	0.73	0.75	0.66	0.76	0.70	0.77	0.72	**0.75**
	DMEFNet	0.78	0.81	0.93	0.85	1.00	0.81	0.81	0.78	0.85	0.67	**0.83**

*Bold values represent the average accuracy for multiple subjects*.

Table 1 shows the prediction accuracy of each subject under different movement prediction models. It can be found that the same movement prediction model has a large difference in the accuracy for different subjects, especially the lower limb movement prediction model based on EEG signal, this further supports the large individual differences in EEG signals.

In general, the DMEFNet proposed in this paper can obtain a better lower limb movement prediction performance in the within-subject situation, and its performance is not statistically different from the performance of other contrast movement prediction models (*P*>0.05).

#### 4.3.2. Cross-Subject Classification

In the case of cross-subject, we randomly selected the data of seven subjects to train the model and selected the data of three subjects as the test set. The whole process is repeated five times, producing five different folds.

**Figure 6:**
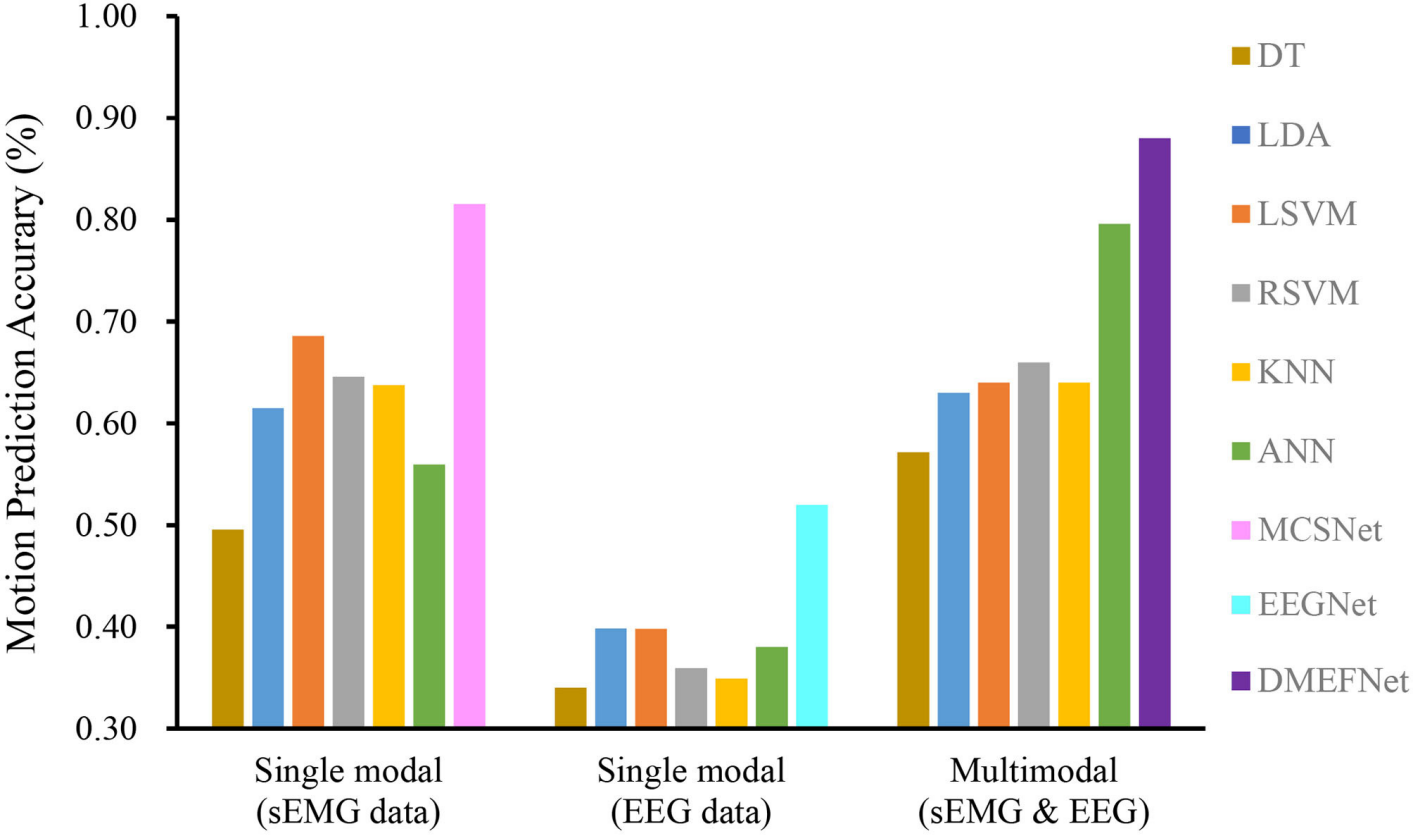
Cross-subject movement prediction performance averaged over all folds. Error bars denote two standard errors of the mean.

Cross-subject prediction results across all models are shown in Figure 6. It can be seen that the traditional and deep learning-based movement prediction models have poor performance in both cases of single modal and multimodal data, which shows that handcraft features cannot effectively characterize the common features of signals of multiple subjects under the same movement. In the case of single modal data, the performance of the movement prediction model based on deep learning is better than that based on traditional machine learning, which shows that the features extracted by the deep learning framework are better than handcrafted features. The important thing is, the DMEFNet model proposed in this paper can achieve the highest accuracy of 88.44% in lower limb movement prediction, which has a significant statistical difference (*P* < 0.05). This means that the DMEFNet proposed in this article can more effectively find the co-attention and mutual mapping relationship between sEMG and EEG signal features, and achieve more accurate lower limb movement prediction.

## 5. Conclusion

In this article, a DCA mechanism based HEI is proposed for lower limb movement prediction in hemiplegia rehabilitation training. The interface constructs a DMEFNet, it uses EEGNet and MCSNet structure to extract the features of EEG and sEMG signals and introduces the DCA mechanism to enhance the common attention between sEMG and EEG signal features, and achieves the deep fusion of multi-modal signal features. An sEMG and EEG data acquisition experiment and an experimental paradigm for the incomplete asynchronous acquisition of EEG and sEMG signals are designed to verify the effectiveness of the DMEFNet model, which solves the problem of large interference to EEG signals when the synchronous acquisition of sEMG and EEG signals of the hemiplegic lower limbs. The experiment results show that DMEFNet has a good movement prediction performance in both within-subject and cross-subject situations. In the future, we consider applying the proposed HEI to an actual exoskeleton platform for rehabilitation training. In addition, We will focus on the study of HEI that can be rapidly adaptive for cross-subject.

## Data Availability Statement

The raw data supporting the conclusions of this article will be made available by the authors, without undue reservation.

## Ethics Statement

The studies involving human participants were reviewed and approved by Ethics Committee of University of Electronic Science and Technology of China. The patients/participants provided their written informed consent to participate in this study. Written informed consent was obtained from the individual(s) for the publication of any potentially identifiable images or data included in this article.

## Author Contributions

KS designed the movement prediction model, performed the experiments, and drafted the manuscript. RH, FM, and CZ participated in the design of the movement prediction model and assisted in the manuscript writing. KH and ZP participated in doing the experiment and implemented the designed algorithm. XY and HC guided the writing of the manuscripts and carrying out experiments. All authors contributed to the article and approved the submitted version.

## Funding

This study was supported by the National Key Research and Development Program of China (no. 2018AAA0102504), the National Natural Science Foundation of China (NSFC) (no. 62003073), the Sichuan Science and Technology Program (nos. 2021YFG0184, 2020YFSY0012, and 2018GZDZX0037), the China Postdoctoral Science Foundation under Grant (nos. 2018M643452 and 2021M700695) and the Research Foundation of Sichuan Provincial People's Hospital (no. 2021LY12).

## Conflict of Interest

The authors declare that the research was conducted in the absence of any commercial or financial relationships that could be construed as a potential conflict of interest.

## Publisher's Note

All claims expressed in this article are solely those of the authors and do not necessarily represent those of their affiliated organizations, or those of the publisher, the editors and the reviewers. Any product that may be evaluated in this article, or claim that may be made by its manufacturer, is not guaranteed or endorsed by the publisher.
